# Distinct CSF α-synuclein aggregation profiles associated with Alzheimer's disease phenotypes and MCI-to-AD conversion

**DOI:** 10.1016/j.tjpad.2024.100040

**Published:** 2025-01-03

**Authors:** Yanfei Ding, Lingbing Wang, Jun Liu, Yulei Deng, Yang Jiao, Aonan Zhao

**Affiliations:** a Department of Neurology and Institute of Neurology, Ruijin Hospital affiliated to the Shanghai Jiaotong University School of Medicine, Shanghai 200025, China; b The ADNI is detailed in Supplemental Acknowledgments

**Keywords:** Alzheimer's disease, Mild cognitive impairment, α-Synuclein, Cerebrospinal fluid

## Abstract

**Background:**

α-Synuclein (α-Syn) pathology is present in 30–50 % of Alzheimer's disease (AD) patients, and its interactions with tau proteins may further exacerbate pathological changes in AD. However, the specific role of different aggregation forms of α-Syn in the progression of AD remains unclear.

**Objectives:**

To explore the relationship between various aggregation types of CSF α-Syn and Alzheimer's disease progression.

**Design:**

We conducted a retrospective analysis of data from the Alzheimer's Disease Neuroimaging Initiative (ADNI) to examine the association between different α-Syn aggregation forms—Syn0 (no detectable α-Syn aggregates) and Syn1 (α-Syn aggregates detected, resembling those found in Parkinson's disease)—with the pathological and clinical features of AD. Additionally, we evaluated their potential as predictors of conversion from mild cognitive impairment (MCI) to AD.

**Setting:**

The ADNI database.

**Participants:**

A total of 250 participants, including 70 cognitively normal controls, 119 patients diagnosed with MCI, and 61 patients diagnosed with AD.

**Measurements:**

Pearson correlation was employed to assess the relationship between α-Syn levels and cerebrospinal fluid (CSF) biomarkers, including total tau (T-tau), phosphorylated tau (p-tau), and amyloid-β_42_ (Aβ_42_). Multivariate Cox proportional hazards models were applied, adjusting for APOE4 status, age, and sex, to determine the association between α-Syn forms and AD-related pathological and clinical outcomes. Kaplan-Meier curves were used to evaluate the prognostic value of different α-Syn aggregation states in predicting the conversion from MCI to AD.

**Results:**

Compared with controls, overall MCI and AD patients had elevated α-Syn levels. Notably, in the α-Syn0 group, α-Syn levels were increased in the MCI patients and further increased in AD patients, whereas in the α-Syn1 group, α-Syn levels did not significantly differ across diagnostic groups. Both in the α-Syn0 and α-Syn1 groups, α-Syn levels were found to correlate more strongly with CSF tau levels than with Aβ_42_, indicating a possible role for α-Syn in tau-related pathology in AD. Importantly, α-Syn0-AD patients exhibited more rapid cognitive decline and greater hippocampal atrophy than α-Syn1-AD patients. However, MCI patients with CSF α-Syn1 aggregation status had an increased risk of conversion to AD.

**Conclusions:**

CSF α-Syn is associated with tau pathology and neurodegeneration in Alzheimer's disease. The distinct aggregation profiles of α-Syn serve as valuable biomarkers, offering insights into differing prognoses in AD and aiding in the prediction of early disease progression.

## 1 Introduction

α-Synuclein (α-Syn) is abundantly present in the brain, primarily existing in its soluble natural form as an unstructured monomer located mainly at presynaptic terminals [1]. α-Syn monomer association with the distal reserve pool of synaptic vesicles and the deficiencies in synaptic transmissions observed in response to knockdown or overexpression of α-syn suggest that α-syn has a role in the regulation of neurotransmitter release, synaptic function and plasticity [2,3]. In contrast to the physiological conformations described above, α-Syn allows misfolded α-Syn conformational isomers to persist and aggregate into pathogenic assemblies under pathological conditions, an important pathological process in degenerative diseases such as Parkinson's disease(PD) and Lewy body dementia(DLB) [4]. High levels of the soluble oligomeric form α-Syn are considered an important pathogenic species, causing cytoskeletal alterations, membrane permeabilisation, increased production of reactive oxygen species, and synaptic toxicity [[5], [6], [7]]. Thus, the apparent versatility of α-Syn may stem from its conformational flexibility.

The main features of Alzheimer's disease (AD) are β-amyloid (Aβ) plaques and tau neurofibrillary tangles [8], however, it is noted that 30–50 % of AD patients also exhibit signs of α-Syn pathology [9]. Patients with autopsy-confirmed Lewy body variants of AD tend to experience more rapid cognitive decline and higher mortality rates [10]. Interestingly, recent research has reported faster and more pronounced cognitive dysfunction with co-expression of tau with α-Syn in the gut, suggesting that the interaction between α-syn and tau may further influence pathological changes in AD [11]. The potential role of cerebrospinal fluid (CSF) α-Syn in the progression of dementia is not fully understood, and studies on its effects on cognition have yielded conflicting results [[12], [13], [14]]. We speculate that functional differences arising from the structural heterogeneity of α-Syn may contribute to the variability in observed outcomes.

Most current studies focused on total or aggregated α-Syn, often overlooking the role of soluble α-Syn and giving little attention to the differences in the functions of various conformational states of α-Syn in AD [15]. A study found that, despite the absence of Lewy body-related pathology, levels of soluble α-Syn were elevated in the inferior temporal lobe of the AD brain [16,17]. Therefore, when analyzing the role of CSF α-Syn in AD, it is essential to differentiate between its various conformational forms. Previous studies have shown that the concentration of CSF α-Syn changes dynamically during AD progression and peaks at the early symptomatic stage [18,19], suggesting that the early increase in CSF α-Syn could be used to predict the mild cognitive impairment (MCI) to AD conversion [20,21]. Consequently, there exists a gap in understanding the predictive capacity of the different structural forms of α-Syn for this conversion.

Hence, in our study, for the first time, we focused on the correlation of different aggregation types of CSF α-Syn with AD-associated pathological proteins and their differences in the phenotypes of AD clinical features. When aggregated forms of CSF α-Syn are detected, it suggests that α-Syn is misfolded in the CSF. We stratified the subjects based on the α-Syn seed amplification assay (SAA) combined with α-syn-Protein misfolding cyclic amplification (PMCA) assay results and categorized patients with no detectable CSF α-Syn aggregates into the Syn0 group, and those with detectable CSF α-Syn aggregates consistent with PD into the Syn1 group (hereafter, the two CSF α-Syn aggregation states will be referred to as α-Syn0 and α-Syn1, respectively). Moreover, to explore the potential role of different conformational states of CSF α-Syn in MCI-to-AD conversion, we further investigated the predictive ability of CSF α-Syn for MCI transformation in α-Syn0 and α-Syn1 groups.

## 2 Materials and methods

### 2.1 Setting and study participants

Primary analyses in the current study utilized data from the ADNI database enrolled 70 healthy controls (HCs), 119 individuals diagnosed with MCI, and 61 individuals diagnosed with AD. All subjects included were performed the assessments of the Mini-Mental State Examination (MMSE), including Montreal Cognitive Assessment (MoCA), Functional Activities Questionnaire (FAQ) scores, Alzheimer's Disease Assessment Scale Cognition 13-item scale (ADAS13), and APOE4 allele carrier status. The study enrolled individuals defined as MCI or AD who adhered to the Mayo Clinic criteria or the criteria outlined in the National Institute of Neurological Disorders and Stroke-Alzheimer Disease and Related Disorders guidelines. Levels of Aβ_42_, P-tau, T-tau, and α-Syn in CSF were quantified in all the individuals. For the data analysis, we cross-sectionally analyzed the levels of α-Syn and Aβ_42_, P-tau, T-tau, and α-Syn in the CSF in individuals diagnosed with MCI. Moreover, we evaluated our hypothesis in individuals classified as having AD according to the newly established ATN framework developed by the National Institute on Aging and Alzheimer's Association.

### 2.2 Magnetic resonance imaging (MRI) assessment

Longitudinal Hippocampal volume was measured to assess neurodegeneration using 3T MPRAGE MRI scans using the ADNI imaging core at UCSF and obtained from the ADNI database (http://adni.loni.usc.edu/) based on FreeSurfer. The median image was created for each participant by accurately registering their images from all longitudinal time points, producing an impartial template specific to each participant. The template was then used to initialize subsequent algorithms, which included surface reconstruction, nonlinear spatial normalization to an atlas space, and parcellation. This strategy ensured a consistent approach to handling images from all time points, minimizing the risk of bias related to temporal order. Before hippocampal volume extraction, we subjected all images to extensive preprocessing. This involved intensity normalization, elimination of non-brain voxels, affine registration to the Talairach space, and segmentation of subcortical white matter and nuclei, followed by a second intensity normalization. Subsequently, we performed surface reconstruction for all images using a series of steps, including nonlinear registration of individual surface models to a spherical atlas and automated brain region parcellation. Comprehensive explanations of the longitudinal FreeSurfer-based imaging pipelines utilized on the ADNI data can be accessed online at http://adni.loni.usc.edu/ and in prior publications.

### 2.3 α-Syn seed amplification assay (αS‐SAA)

*The dataset of α-Syn levels was obtained from the ADNI database (http://adni.loni.usc.edu/). The assessment of synuclein seeds in CSF was conducted using the synuclein seed amplification assay offered by Amprion (https://ampriondx.com/). The α-Syn seed amplification assay took place in the Amprion Clinical Laboratory (CLIA ID No. 05D2209417; CAP No. 8,168,002) and followed a validated method approved for clinical use in compliance with Clinical Laboratory Improvement Amendment (CLIA) requirements. Each sample is analyzed in triplicate in a 96-well plate using a reaction mixture comprised of 100mM PIPES* pH *6.5, 0.5* *M NaCl, 0.1 % sarkosyl, 10* *µM ThT, 0.3mg/mL recombinant α-Syn, and 40* *µL CSF, in a final volume of 100µL. Two silicon nitride beads are included in each well, and positive and negative assay quality control samples are included on each plate. Plates are sealed with optical adhesive film, placed into the chamber of a BMG LABTECH FLUOstar Ω Microplate Reader, and incubated at 42* °*C with cycles of 1* min *of shaking followed by 14 min of rest with fluorescence measured after every shaking cycle (excitation wavelength 440* *nm, emission 490* *nm). After a total incubation time of 20 h, the maximum fluorescence for each well is determined and an algorithm was applied to the triplicate determinations for each sample for result classification. Patients with negative αS‐SAA results were categorised into the α-Syn0 group.*

### 2.4 α-syn-Protein misfolding cyclic amplification (PMCA)

*The dataset of PMCA results for CSF α-Syn was obtained from the ADNI database (http://adni.loni.usc.edu/). The α-Syn-PMCA assay was performed as previously described [**22**]. In brief, samples of seed-free, monomeric α-syn at a concentration of 1mg/ml in 100mM PIPES,* pH *6.5 and 500* *mM NaCl were placed in opaque 96-well plates (Costar, REF 3916) in the presence of 5 μM ThT at a final volume of 200 μl. For each test, we added 40 μl of CSF from patients and controls or 40 μl of brain homogenate (at a final concentration of 0.001 %). Positive controls consisted of a well-documented and previously screened healthy CSF sample spiked with preformed α-Syn oligomeric seeds. Samples were subjected to cyclic agitation (1* min *at 500* *rpm followed by 29* min *without shaking) at 37 * °*C. The increase in ThT fluorescence was monitored at an excitation of 435* *nm and emission of 485* *nm, periodically, using a microplate spectrofluorometer Gemini-EM (Molecular Devices). Samples with maximum fluorescence values between 2000 and 8000 units were identified as having aggregated α-Syn consistent with the aggregation state in PD [**22**], and these patients were classified as being in the α-Syn1 group.*

### 2.5 Measurements of α-Syn and AD biomarkers

*The measurements of CSF* Aβ_42_*, T-tau, and P-tau within the ADNI database were conducted by the University of Pennsylvania's ADNI Biomarker Core. These measurements employed the multiplex xMAP Luminex platform (Luminex Corp, Austin, TX, USA) in conjunction with the INNOBIA AlzBio3 assay kit (Fujirebio, Ghent, Belgium). For the determination of total CSF α-Syn levels, the Luminex MicroPlex Microspheres (Luminex Corp, Austin, TX) were used, alongside a biotinylated goat anti-human α-synuclein antibody provided by R&D Systems (catalog # BAF1338) [**23**]. To ensure the accuracy and consistency of the results, all samples, along with six CSF standards per plate, were processed in duplicate and normalized against the values obtained from the CSF standards [**24**].*

### 2.6 Statistical analysis

Continuous baseline variables were presented as mean (SE) and discrete variables were summarized as frequencies and percentages. For comparisons of groups of continuous-type variables, after passing the normality test and or the variance chi-square test, the ANOVA or the Kruskal Wallis, the *t*-test or the nonparametric test were used where appropriate. The chi-square test was employed as appropriate for comparisons of Discrete variables. Correlations were corrected by multiple comparisons. All biomarker variables were included alongside variables from the reference model, including age, sex, years of education, and ApoE ε4 carrier status, each independently assessed. The model performance was evaluated using the receiver operating characteristic (ROC) area under the curve (AUC) estimation. The cutoff values were determined using ROC analysis, considering AUC, sensitivity, and specificity. The optimal cutoff point was the value that minimized the sum of absolute differences between the AUC and sensitivity or specificity, ensuring minimal disparity between sensitivity and specificity. Pearson's correlation test was applied to establish associations between α-Syn and T-tau, p-tau, and Aβ_42_ in the CSF while accounting for other covariates. Multivariable analyses, adjusted for APOE4, age, and sex, were performed using the Cox proportional hazards model to gauge the risk of AD progression. A significance level of *P* < 0.05 was the threshold for all statistical tests. Statistical analyses were conducted using SPSS, version 20 (IBM Corp., Armonk, NY, USA) and R, version 4.2.1 (R Development Core Team, Vienna, Austria).

## 3 Results

### 3.1 Demographic and clinical characteristics of participants in baseline

**Fig. 1:**
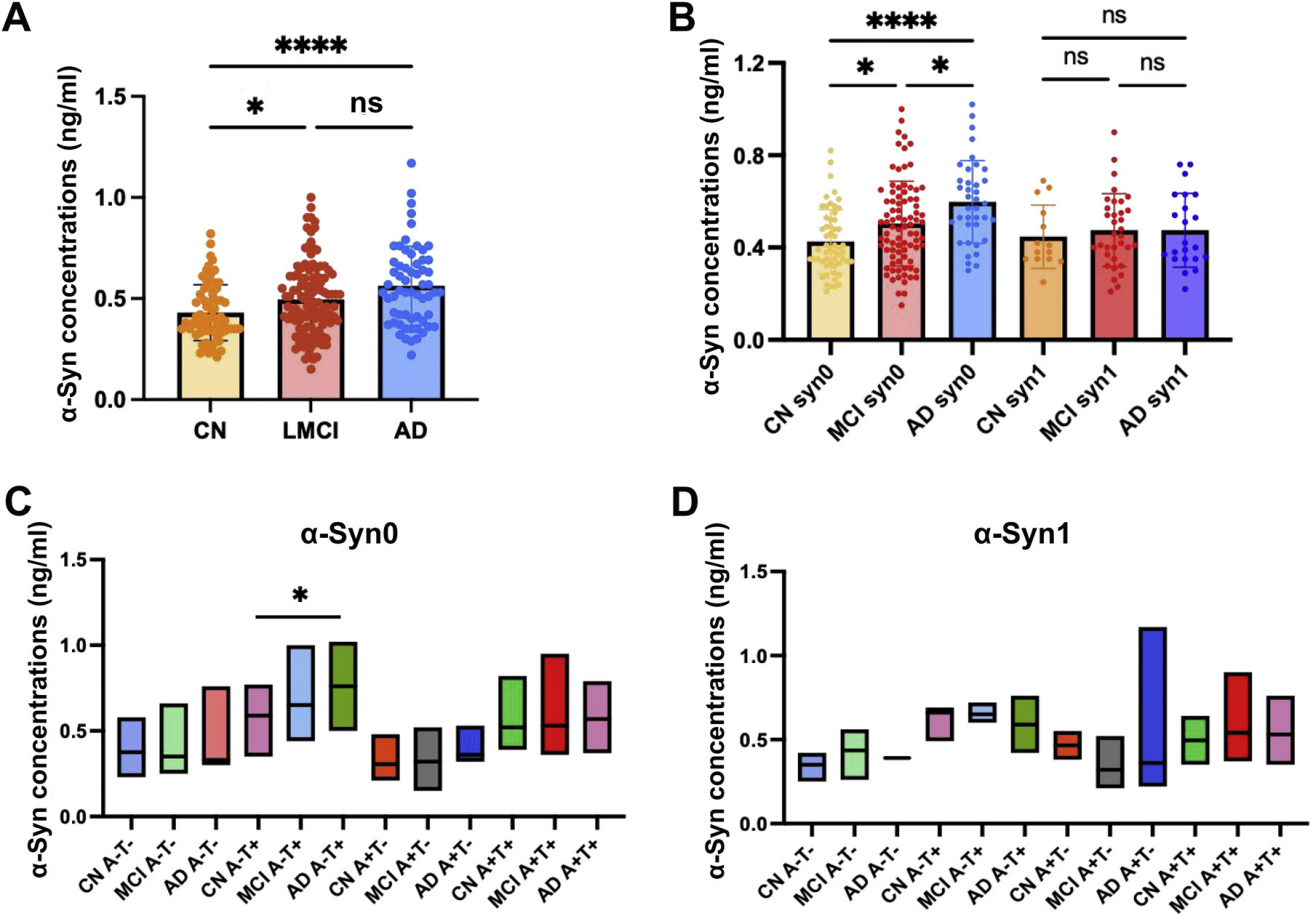
**CSF α-Syn levels in MCI and Alzheimer's disease cohort. A, B:** Quantification of CSFα-Syn levels in the whole group (A), in the α-Syn0 and α-Syn1 group (B) at baseline in different diagnostic groups. **C, D:** Analysis of CSF α-Syn levels in the α-Syn0 (C) and CSF α-Syn1 (D) group between different subgroups based on the ATN framework. *P < 0.05, by one-way ANOVA with the least significant difference post hoc test.

Participants were 119 patients with MCI, 61 patients with AD, and 70 controls. The demographic characteristics, clinical features, and CSF biomarker levels of all participants are reported in Table 1. There were no differences in age (*p* = 0.376), gender (*p* = 0.052), and education duration (*p* = 0.588) among the three groups. As expected, MMSE, FAQ, CSF Aβ_42_, CSF p-tau, CSF T-tau, and APOE ε4 differed between the diagnostic groups at baseline. Similarly to previous studies of the ADNI cohort,α-Syn levels increased significantly in MCI and AD patients (Fig. 1A). Subsequently, subjects were divided into α-Syn0 and α-Syn1 subgroups based on the differences in CSF α-Syn aggregation status (see the method section for details).

**Table 1:** Demographic features of the participants in baseline.

**Demographics**	**CN** **(*n*=70)**	**MCI** **(*n*=119)**	**AD** (*n* = 61)	**p value**
Age(y)	75.42 (4.96)	73.92 (7.51)	74.51 (8.36)	0.252
Gender				
Female	35	39	27	0.052
Male	35	80	34	
Education duration(y)	15.86 (2.48)	15.56 (2.95)	15.34 (3.10)	0.588
ApoE ε4 carrier				
(+)	14 (20.00 %)	65 (54.62 %)	38 (62.30 %)	**<0.0001**
(-)	56 (80.00 %)	54 (45.38 %)	23 (37.70 %)	
MMSE	29.07 (1.08)	26.92 (1.75) *	23.77 (1.90) *^#^	**<0.0001**
FAQ	0.20 (0.79)	4.70 (4.68) *	12.77 (6.26) *^#^	**<0.0001**
CSF Aβ_42_(pg/ml)	1188 (437.40)	817 (420.80) *	691.40 (345.10) *	**<0.0001**
CSF p-tau (pg/ml)	21.51 (8.29)	31.01 (15.01) *	35.64 (16.26) *	**<0.0001**
CSF t-tau (pg/ml)	236 (81.70)	311.20 (130.20) *	351.30 (136.40) *	**<0.0001**
CSF α- synuclein				
1	14 (20.00 %)	34 (28.57 %)	23 (37.70 %)	0.081
0	56 (80.00 %)	85 (71.43 %)	38 (62.30 %)	

**Abbreviations:** MMSE: Mini Mental State Examination; FAQ: functional activities questionnaire; CN: Cognitive Normal; MCI: mild cognitive impairment; AD: Alzheimer's disease. *: Compared with CN group, #: Compared with MCI group.

### 3.2 Differences in levels of different aggregation types of CSF α-Syn between diagnostic groups

After stratifying subjects according to α-Syn aggregation status, we found that in the α-Syn0 group, α-Syn levels were significantly increased in MCI patients and further increased in AD patients (Fig. 1B). However, no significant differences in α-Syn concentration were observed between the diagnostic groups at baseline in the α-Syn1 group (Fig. 1B). Next, we analyzed the differences in α-Syn levels between the CSF α-Syn0 and α-Syn1 groups across diverse pathological subgroups. According to the ATN framework, participants were classified into Aβ positive (*A*+) and Aβ negative (A-) or tau positive (*T*+) and tau negative (T-) categories based on the neuropathological findings. In the α-Syn1 group, the differences in the α-Syn concentration among the subgroups of HC, MCI, and AD were not statistically significant (Fig. 1D). Nevertheless, in the A-*T*+ subgroups of the α-Syn0 group, we found an increasing trend of CSF α-Syn concentration in MCI and AD patients and significant differences between AD and HC (Fig. 1C). These results suggest that CSF α-Syn level may be associated not only with the disease state but also with other pathological markers.

### 3.3 Correlation of concentrations of different aggregation types of CSF α-Syn with Aβ and tau pathology levels

**Fig. 2:**
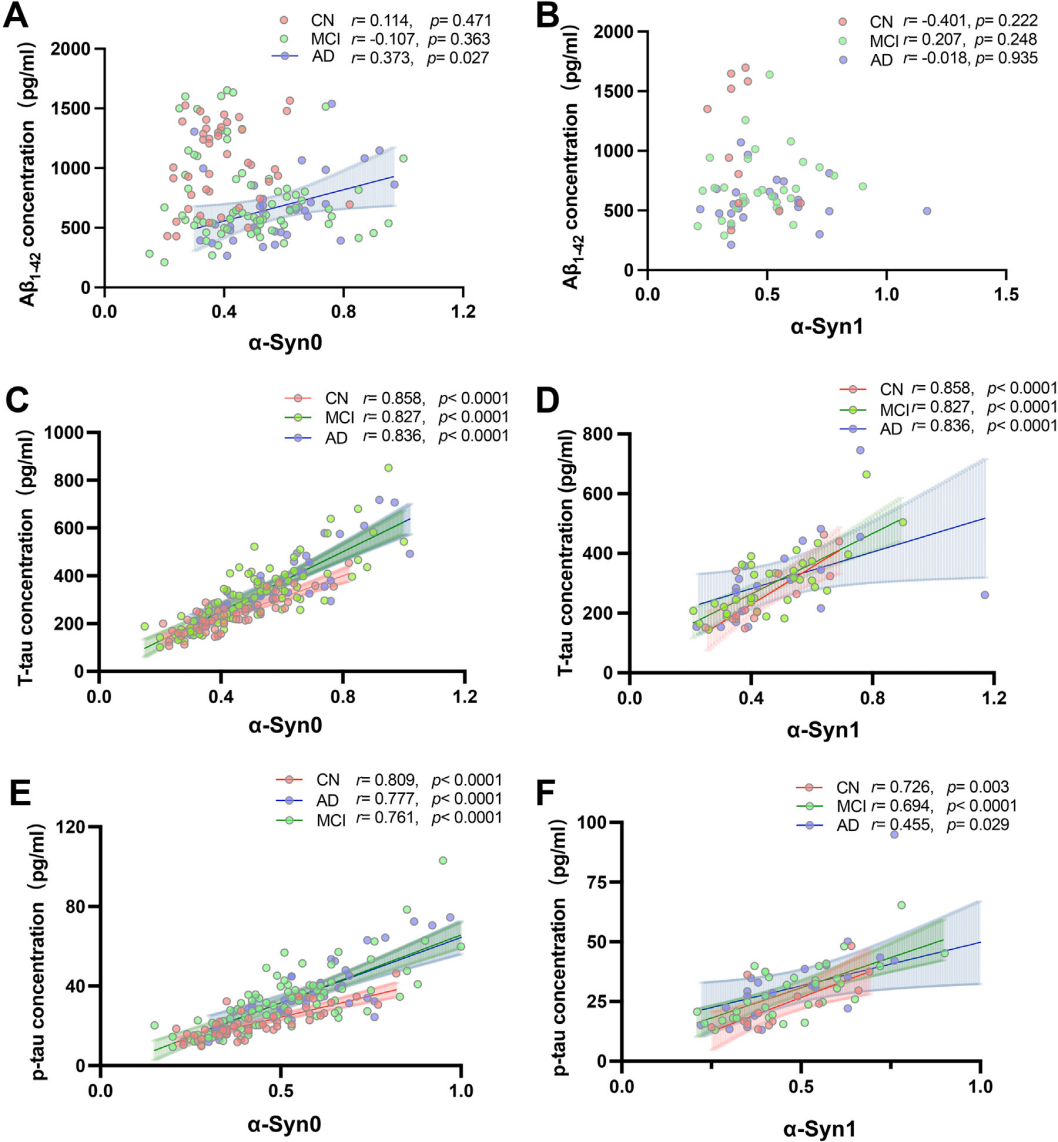
**Association between CSF α-Syn levels and CSF Aβ and tau pathology levels. A, B:** Association between CSF α-Syn and Aβ_42_ levels in the α-Syn0 (A) and α-Syn1 (B) group. **C, D:** Association between CSF α-Syn and T-tau levels in the α-Syn0 (C) and α-Syn1 (D) group. **E, F:** Association between CSF α-Syn and p-tau levels in the α-Syn0 (E) and α-Syn1 (F) group. *P < 0.05, by one-way ANOVA with the least significant difference post hoc test and Pearson's correlation test.

We used CSF Aβ_42_, T-tau, and p-tau levels as pathological indicators of AD. Except in the α-Syn0 group, for the weak association between CSF α-Syn and Aβ_42_ observed in AD patients, there was no other significant correlation between α-Syn concentrations and Aβ_42_ level (Fig. 2A-B). However, in the α-Syn0 group, a strong correlation was observed between α-Syn and both CSF T-tau and p-tau levels (Fig. 2C, 2E). In the α-Syn1 group, a positive correlation of α-Syn concentration with CSF T-tau and p-tau was also observed (Fig. 2D, 2F). These findings suggest that α-Syn may be more closely linked to tau-related AD pathology and progression, rather than Aβ_42_.

### 3.4 Effect of concentrations of different aggregation types of CSF α-Syn on cognition decline in individuals with AD

**Fig. 3:**
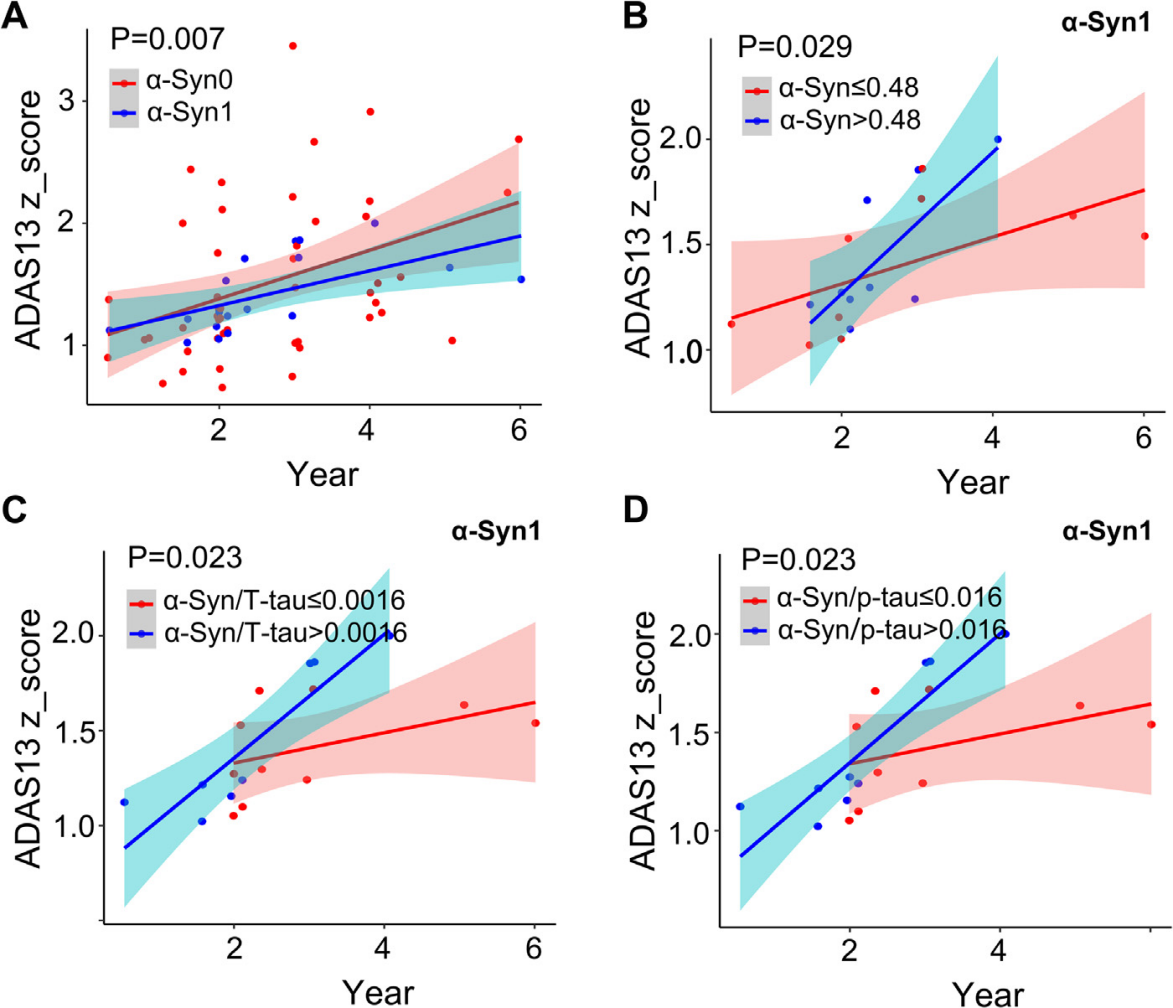
**Correlation analysis of CSF α-Syn with cognitive decline in AD patients. A:** Patients in the α-Syn0 group experienced significantly faster cognitive decline than those in the α-Syn1 group. **B:** In the α-Syn1 group, patients with CSF α-Syn levels higher than 0.48 ng/ml experienced faster cognitive impairment. **C:** In the α-Syn1 group, patients with CSF α-Syn/T-tau ratio higher than 0.0016 experienced faster cognitive impairment. **D:** In the α-Syn1 group, patients with CSF α-Syn/p-tau ratio higher than 0.016 experienced faster cognitive impairment. The cut-off values were determined by using the ROC curve with Youden's index. Multivariable analyses with the Cox proportional hazards model.

Then we investigated the impact of different types of CSF α-Syn structural states on cognitive decline in the follow-up of AD groups. Cognitive changes were assessed using the ADAS13 score, adjusting for sex, age, education, and ApoE ε4 carrier status. Patients in the α-Syn0 group experienced significantly faster cognitive decline than those in the α-Syn1 group (Fig. 3A). Then α-Syn levels were categorized as below or above the cut-off value. The cut-off values were determined using the ROC curve with Youden's index. The determined cut-off value for α-Syn was 0.48. In the α-Syn1 group, higher α-Syn concentrations were associated with a faster rise in ADAS13 scores, consistent with the results observed in the overall α-Syn group but not with those in the α-Syn0 subgroup (Fig. 3B, Supplement Figure 1A, 1D). Due to the significant correlation between α-Syn and tau pathology, we calculated the ratio of α-Syn to tau. The ROC curve determines the cut-off value of α-Syn/T-tau and α-Syn/p-tau to be 0.0016 and 0.016 respectively. We observed results in the α-Syn1 subgroup that were consistent with those in the overall α-Syn group. Higher α-Syn/T-tau and α-Syn/p-tau ratios were associated with a faster rise in ADAS13 scores, implying more rapid cognitive decline (Fig. 3C-D, Supplement Figure 1B-C). In contrast, these results were not observed in the α-Syn0 subgroup. (Supplement Figure 1E-F). In summary, patients in the α-Syn0 group experienced faster cognitive decline than those in the α-Syn1 group. Meanwhile, in the α-Syn1 group, high concentrations of CSF α-Syn contributed to more pronounced cognitive decline.

### 3.5 Correlation of the level of different types of α-Syn structural states with hippocampal volume changes in AD patients

**Fig. 4:**
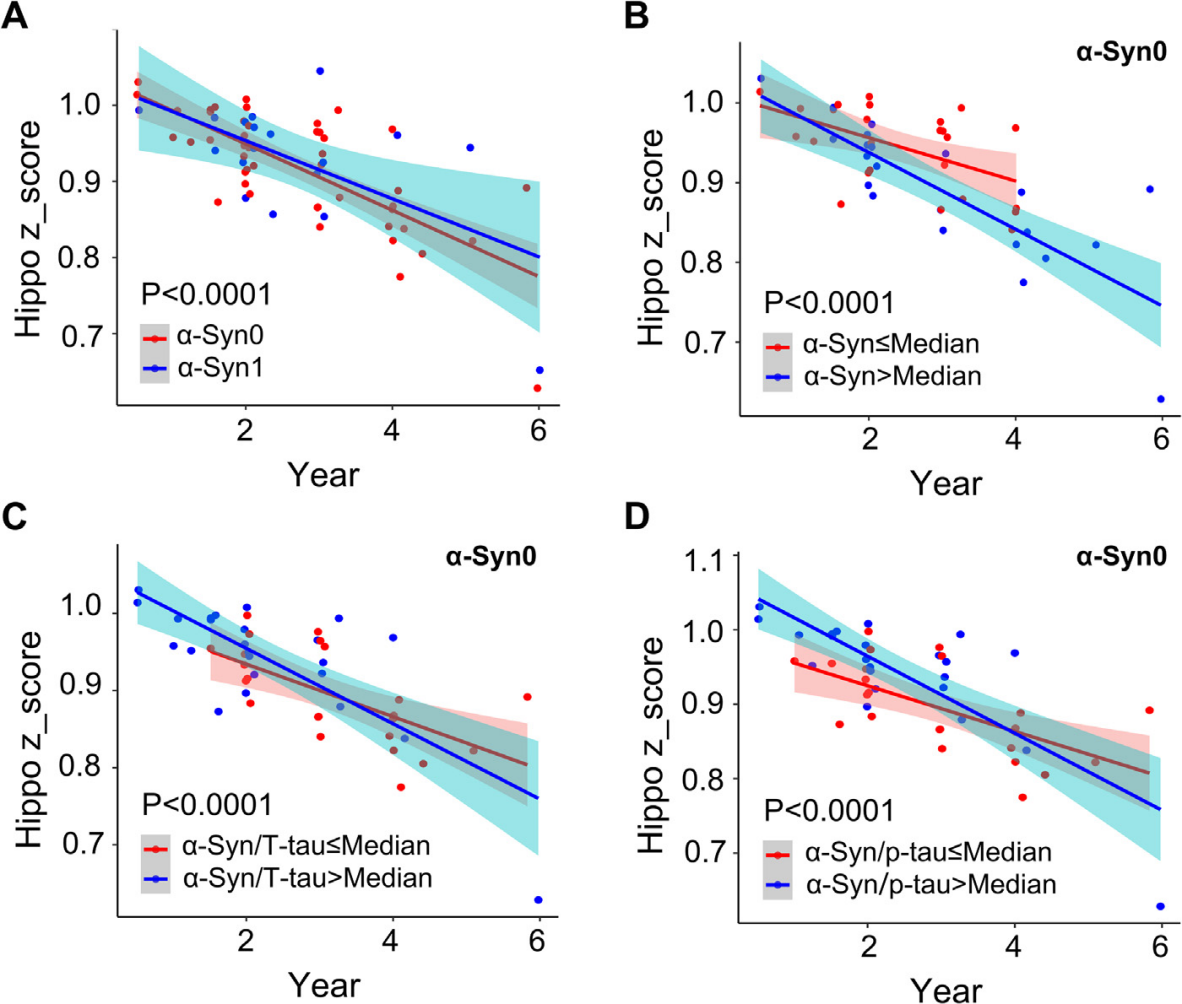
**Correlation analysis of CSF α-Syn with hippocampal volume changes in AD patients. A:** Patients in the α-Syn0 group had a faster decline in hippocampal volume compared to patients in the α-Syn1 group. **B:** In the α-Syn0 group, a high level of α-Syn was associated with a faster decline in hippocampal volume. **C:** In the α-Syn0 group, a high ratio of CSF α-Syn/T-tau was associated with decreased hippocampal volume. **D:** In the α-Syn0 group, a high ratio of CSF α-Syn/p-tau was associated with decreased hippocampal volume. Multivariable analyses with the Cox proportional hazards model.

To assess the impact of α-Syn levels on the progression of cognition decline, we analyzed the correlation between α-Syn levels and changes in hippocampal volume in follow-up visits of the AD group. All results were adjusted for sex, age, education, and ApoE ε4 carrier status. We found that AD patients in the α-Syn0 group had a faster decline in hippocampal volume compared to patients in the α-Syn1 group (Fig. 4A). α-Syn levels were dichotomized into high and low groups based on the median expression as a cutoff value. As shown in Fig. 4B-D, in the α-Syn0 group, higher α-Syn concentrations, higher ratios of α-Syn/ T-tau, and α-Syn/ p-tau were associated with faster hippocampal volume decline (Fig. 4B-D), which is consistent with the results observed in the overall α-Syn group (Supplement Figure 2A-C). However, in the α-Syn1 group, no correlation was found between α-Syn concentration and changes in hippocampal volume (Supplement Figure 2D-F).

### 3.6 Correlation between α-Syn aggregation type and MCI conversion

Since CSF α-Syn0 and α-Syn1 levels were associated with tau pathology and therefore could be predictive of disease progression. Of 82 patients who were followed up, a total of 48(58.5 %) developed definite AD (Supplemental Table 1). We found no significant differences in age, sex, ApoE ε4–carrier genotype, or years of education between the groups.

**Fig. 5:**
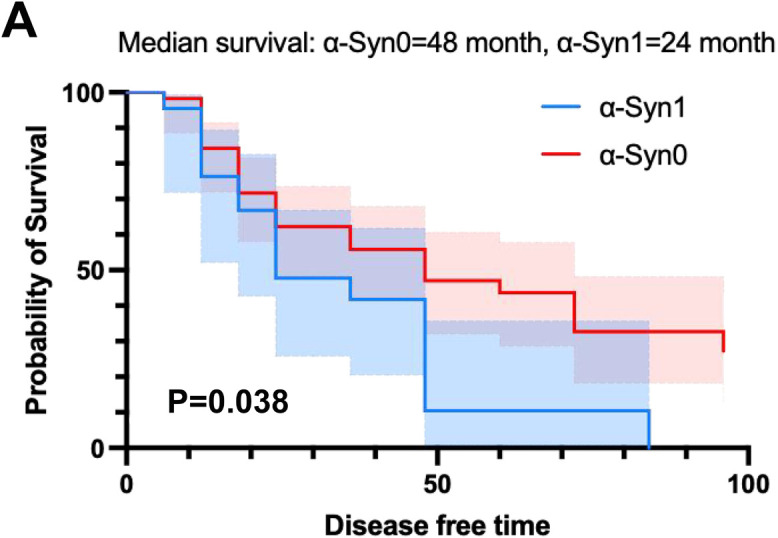
**Kaplan-Meier plot of AD-free survival with MCI with different CSF α-Syn aggregation types (α-Syn0 and α-Syn1).** Data were analyzed using the Kaplan-Meier method.

Furthermore, we applied Cox analysis to evaluate whether each CSF biological level could predict conversion after adjusting for age, and sex. The results showed that patients with MCI who had CSF α-Syn1 (HR = 2.890, *P* = 0.040, Supplemental Table 2), CSF Aβ_42_ levels higher than 730.0 pg/mL (HR = 2.575, *P* = 0.045, Supplemental Table 2), CSF p-tau levels higher than 29.0 pg/mL (HR = 4.300, *P* = 0.002, Supplemental Table 2), and CSF T-tau levels higher than 310.0 pg/mL (HR = 3.132, *P* = 0.017, Supplemental Table 2) were more likely to develop AD. Kaplan-Meier curves were used to evaluate the predictive value of CSF α-Syn levels in different aggregation states in the conversion process. Patients with MCI in the α-Syn1 group, had a mean AD-free survival of 24 months, whereas those in α-Syn0 group had a mean AD-free survival of 48 months (*P* = 0.0376, Fig. 5A). Furthermore, patients with MCI who had CSF α-Syn1 aggregation type were more likely to develop AD.

## 4 Discussion

Previous studies have shown conflicting results regarding the potential role of CSF α-Syn in AD and its effect on cognitive performance, which may be due to the structural heterogeneity of α-Syn. Therefore, in the present study, for the first time, we stratified subjects according to the aggregation status of CSF α-Syn (α-Syn0: α-Syn aggregates not detected, α-Syn1: α-Syn aggregates detected, aggregation profile consistent with that in PD) and compared α-Syn levels and their correlation with AD pathology in AD, MCI and healthy controls. In addition, we analyzed the correlation between α-Syn level and clinical phenotypes of AD, including cognitive decline and hippocampal atrophy. Moreover, for the first time, we analyzed and compared the predictive ability and differences between different aggregation types of CSF α-Syn for the conversion of MCI to AD. We found that compared to CN, α-Syn levels were significantly increased in MCI and AD patients. However, in the α-Syn0 group, compared to controls, α-Syn levels were significantly increased in MCI patients and further increased in AD patients, whereas in the α-Syn1 group, α-Syn levels did not differ between diagnostic groups. This partly explains the inconsistent correlation between CSF α-Syn and AD in some studies, and we discuss the possible clinical significance of these results later. It also suggests the necessity to explore the role of different aggregation types of α-Syn in AD.

Abnormal aggregation of tau protein is one of the core pathological features of AD, while the accumulation of Aβ plaques, though also associated with AD, does not show a significant correlation with α-Syn levels in this result. In the present study, we found that both in the α-Syn0 and α-Syn1 group, α-Syn levels correlated with T-tau and p-tau levels in CSF but showed no significant association with Aβ levels. We speculate that α-Syn may be closely associated with tau pathology in AD compared to Aβ. NMR imaging revealed that the monomeric form of tau selectively interacted with the C-terminal region of the α-Syn monomer and accelerated α-Syn oligomerization and subsequent fibril formation [25]. Besides, the dependence of tau phosphorylation on the presence of α-Syn was demonstrated in α-Syn-deficient cell and mouse models [26,27], indicating α-Syn monomers and fibrils promoted tau aggregations [25]. This evidence for α-Syn-mediated accumulation of tau content and phosphorylation suggests that under pathophysiological conditions, elevated α-Syn may encourage phosphorylation tau, leading to tau pathology through significantly elevated phosphorylated tau. All this evidence indicates that α-Syn may influence tau pathology through its interaction with tau protein. In addition, a recent study found that CSF α-Syn-positive conversion was associated with CSF Aβ_42_ positivity, highlighting the interaction between Aβ and α-Syn [28]. The current findings indicated that α-Syn may play a pivotal role in tau pathology in AD, with a comparatively limited involvement in Aβ pathology [29]. This differential association provides critical insights into the distinct pathological mechanisms of AD.

Furthermore, we analyzed in depth the correlation between the levels of CSF α-Syn in different aggregation states and cognitive decline and hippocampal atrophy. Our results show that cognitive impairment and hippocampal atrophy are faster in CSF α-Syn0-AD patients than in α-Syn1-AD patients. Interestingly, in the α-Syn1 subgroup, we found that high levels of α-Syn caused faster cognitive decline without the correlation in hippocampal atrophy. In contrast, in the α-Syn0 group, high levels of α-Syn caused faster changes in hippocampal atrophy without detectable correlation with changes in cognitive impairment. Considering the high correlation between tau and α-Syn levels, abnormal neuronal synaptic function followed by disruption may increase the release of α-Syn into the cerebrospinal fluid, similar to the release of tau from neuronal death [30]. In support, CSF α-Syn levels were significantly elevated in patients with AD with all positive CSF triple markers (Aβ_42_, total tau, and phosphorylated tau) [15,31]. Therefore, we speculate that dysregulation of CSF α-Syn levels in the CSF α-Syn0 group may represent a critical neurobiological event associated with the disruption of synaptic function that occurs during AD physiopathology. Elevated CSF α-Syn levels in the α-Syn0 group may signal the rapid progression of AD, as confirmed by significantly higher α-Syn levels in the α-Syn0-AD patients at baseline, which needs to be followed up with further validation discussions.

Numerous studies have reported α-Syn pathology in approximately 50–60 % of autopsied AD patients [[32], [33], [34]]. Patients with mixed pathologies as well as animal models expressing combined AD and α-Syn pathologies tend to exhibit earlier cognitive impairment [35,36]. Conversely, up to 50 % of PD patients with dementia patients develop Aβ and tau pathology sufficient for a secondary pathological diagnosis of AD [37,38]. The results of these studies suggest that α-Syn pathology may be involved early in AD pathogenesis and is associated with the progression of cognitive dysfunction. Patients with CSF α-Syn1 were more likely to develop Lewy body pathology. In the α-Syn1 group, the correlation between high levels of CSF α-Syn and increased ADAS13 scores supports this conjecture. We further speculate that α-Syn levels may be critical in the early diagnosis and identification of preclinical AD for optimizing patient outcomes in the α-Syn1 group. Therefore, we analyzed and compared the roles of different CSF α-Syn aggregation states in the MCI to AD conversion process. We found that α-Syn1-MCI patients had a mean AD-free survival of 24 months, whereas α-Syn0-MCI patients had a mean AD-free survival of 48 months. These results provide evidence for the predictive value of CSF α-Syn1 aggregation states in the transition from MCI to AD and suggest that CSF α-Syn1 aggregation states may be involved in the pathophysiological mechanisms underlying the early preclinical onset of AD and warrant further exploration. Interestingly, CSF α-Syn levels were not significantly increased in the CSF α-Syn1-AD patients at baseline, which may confirm that AD primarily involves tau or Aβ pathology and is not a synaptic nucleoprotein disease. Previous studies have suggested that α-Syn is involved in the early generation of Aβ and tau pathology and co-precipitates with it [30], which could explain the faster AD transformation of α-Syn1-MCI. We therefore speculate that the degree of α-Syn1 aggregation state may be associated with the development of AD, which requires further experimental exploration.

In conclusion, we found that CSF α-Syn was associated with AD tau pathology. CSF α-Syn in different aggregation states is associated with different AD clinical phenotypes and early transformation. In the Syn0 group, high concentrations of CSF α-Syn implied faster cognitive impairment with hippocampal atrophy, whereas CSF α-Syn1 aggregation status is associated with more rapid MCI to AD conversion. These results suggest that α-Syn0 and α-Syn1 aggregation status may be biomarkers for rapid progression of AD and the risk of MCI to AD transition, respectively, which may be related to the involvement of different aggregation types of α-Syn in different pathophysiological mechanisms of AD. For the correlation between CSF α-Syn and AD, further in-depth studies are needed to provide more accurate insights into AD and may open new avenues for disease prevention and treatment.

## Disclosure

The authors have declared that no conflict of interest exists.

## CRediT authorship contribution statement

**Yanfei Ding:** Writing – review & editing, Writing – original draft, Data curation. **Lingbing Wang:** Writing – review & editing, Validation. **Jun Liu:** Supervision. **Yulei Deng:** Writing – review & editing, Supervision, Methodology, Conceptualization. **Yang Jiao:** Writing – review & editing, Validation, Supervision, Investigation, Data curation, Conceptualization. **Aonan Zhao:** Methodology, Funding acquisition, Conceptualization.

## Declaration of competing interest

This research was supported for all authors by the 10.13039/501100001809National Natural Science Foundation of China (82,201,392), the Shanghai Sailing Program (22YF1425100), the Alzheimer's Disease Neuroimaging Initiative (ADNI). No other conflicts of interest.

## Appendix Supplementary materials

Image, application 1

## References

[1] Iwai A., Masliah E., Yoshimoto M. (1995). The precursor protein of non-a beta component of alzheimer's disease amyloid is a presynaptic protein of the central nervous system. Neuron.

[2] Cabin D.E., Shimazu K., Murphy D. (2002). Synaptic vesicle depletion correlates with attenuated synaptic responses to prolonged repetitive stimulation in mice lacking alpha-synuclein. J Neurosci.

[3] Lashuel H.A., Overk C.R., Oueslati A. (2013). The many faces of α-synuclein: from structure and toxicity to therapeutic target. Nat Rev Neurosci.

[4] Morimoto R.I. (2011). The heat shock response: systems biology of proteotoxic stress in aging and disease. Cold Spring Harb Symp Quant Biol.

[5] Danzer K.M., Haasen D., Karow A.R. (2007). Different species of alpha-synuclein oligomers induce calcium influx and seeding. J Neurosci.

[6] Parihar M.S., Parihar A., Fujita M. (2009). Alpha-synuclein overexpression and aggregation exacerbates impairment of mitochondrial functions by augmenting oxidative stress in human neuroblastoma cells. Int J Biochem Cell Biol.

[7] Choi B.K., Choi M.G., Kim J.Y. (2013). Large α-synuclein oligomers inhibit neuronal snare-mediated vesicle docking. Proc Natl Acad Sci U S A.

[8] Scheltens P., De Strooper B., Kivipelto M. (2021). Alzheimer's disease. Lancet.

[9] Barnes L.L., Leurgans S., Aggarwal N.T. (2015). Mixed pathology is more likely in black than white decedents with alzheimer dementia. Neurology.

[10] Olichney J.M., Galasko D., Salmon D.P. (1998). Cognitive decline is faster in lewy body variant than in alzheimer's disease. Neurology.

[11] Xiang J., Tang J., Kang F. (2024). Gut-induced alpha-synuclein and tau propagation initiate parkinson's and alzheimer's disease co-pathology and behavior impairments. Neuron.

[12] Van Hulle C., Jonaitis E.M., Betthauser T.J. (2021). An examination of a novel multipanel of csf biomarkers in the alzheimer's disease clinical and pathological continuum. Alzheimers Dement.

[13] Mollenhauer B., Cullen V., Kahn I. (2008). Direct quantification of csf alpha-synuclein by elisa and first cross-sectional study in patients with neurodegeneration. Exp Neurol.

[14] Førland M.G., Tysnes O.B., Aarsland D. (2020). The value of cerebrospinal fluid α-synuclein and the tau/α-synuclein ratio for diagnosis of neurodegenerative disorders with lewy pathology. Eur J Neurol.

[15] Majbour N.K., Chiasserini D., Vaikath N.N. (2017). Increased levels of csf total but not oligomeric or phosphorylated forms of alpha-synuclein in patients diagnosed with probable alzheimer's disease. Sci Rep.

[16] Larson M.E., Sherman M.A., Greimel S. (2012). Soluble α-synuclein is a novel modulator of alzheimer's disease pathophysiology. J Neurosci.

[17] Larson M.E., Greimel S.J., Amar F. (2017). Selective lowering of synapsins induced by oligomeric α-synuclein exacerbates memory deficits. Proc Natl Acad Sci U S A.

[18] Twohig D., Rodriguez-Vieitez E., Sando S.B. (2018). The relevance of cerebrospinal fluid α-synuclein levels to sporadic and familial alzheimer's disease. Acta Neuropathol Commun.

[19] Wang H., Stewart T., Toledo J.B. (2018). A longitudinal study of total and phosphorylated α-synuclein with other biomarkers in cerebrospinal fluid of alzheimer's disease and mild cognitive impairment. J Alzheimers Dis.

[20] Seino Y., Nakamura T., Kawarabayashi T. (2019). Cerebrospinal fluid and plasma biomarkers in neurodegenerative diseases. J Alzheimers Dis.

[21] Shi M., Tang L., Toledo J.B. (2018). Cerebrospinal fluid α-synuclein contributes to the differential diagnosis of alzheimer's disease. Alzheimers Dement.

[22] Shahnawaz M., Mukherjee A., Pritzkow S. (2020). Discriminating α-synuclein strains in parkinson's disease and multiple system atrophy. Nature.

[23] Hong Z., Shi M., Chung K.A. (2010). Dj-1 and alpha-synuclein in human cerebrospinal fluid as biomarkers of parkinson's disease. Brain.

[24] Liu W., Li W., Liu Z. (2024). Cerebrospinal fluid α-synuclein adds the risk of cognitive decline and is associated with tau pathology among non-demented older adults. Alzheimers Res Ther.

[25] Dasari A.K.R., Kayed R., Wi S. (2019). Tau interacts with the c-terminal region of α-synuclein, promoting formation of toxic aggregates with distinct molecular conformations. Biochemistry.

[26] Duka T., Rusnak M., Drolet R.E. (2006). Alpha-synuclein induces hyperphosphorylation of tau in the mptp model of parkinsonism. Faseb J.

[27] Duka T., Duka V., Joyce J.N. (2009). Alpha-synuclein contributes to gsk-3beta-catalyzed tau phosphorylation in parkinson's disease models. Faseb J.

[28] Tosun D., Hausle Z., Thropp P., et al., Association of csf α-synuclein seed amplification assay positivity with disease progression and cognitive decline: a longitudinal alzheimer's disease neuroimaging initiative study. medRxiv. 2024. doi: 10.1101/2024.07.16.24310496. 10.1002/alz.14276 PMC11667524 39428831

[29] Cousins K.A.Q., Arezoumandan S., Shellikeri S. (2022). Csf biomarkers of alzheimer disease in patients with concomitant α-synuclein pathology. Neurology.

[30] Twohig D., Nielsen H.M. (2019). Α-synuclein in the pathophysiology of alzheimer's disease. Mol Neurodegener.

[31] Shim K.H., Kang M.J., Suh J.W. (2020). Csf total tau/α-synuclein ratio improved the diagnostic performance for alzheimer's disease as an indicator of tau phosphorylation. Alzheimers Res Ther.

[32] Arai Y., Yamazaki M., Mori O. (2001). Alpha-synuclein-positive structures in cases with sporadic alzheimer's disease: morphology and its relationship to tau aggregation. Brain Res.

[33] Hamilton R.L. (2000). Lewy bodies in alzheimer's disease: a neuropathological review of 145 cases using alpha-synuclein immunohistochemistry. Brain Pathol.

[34] Lippa C.F., Fujiwara H., Mann D.M. (1998). Lewy bodies contain altered alpha-synuclein in brains of many familial alzheimer's disease patients with mutations in presenilin and amyloid precursor protein genes. Am J Pathol.

[35] Clinton L.K., Blurton-Jones M., Myczek K. (2010). Synergistic interactions between abeta, tau, and alpha-synuclein: acceleration of neuropathology and cognitive decline. J Neurosci.

[36] Mueller C., Ballard C., Corbett A. (2017). The prognosis of dementia with lewy bodies. Lancet Neurol.

[37] Irwin D.J., Lee V.M., Trojanowski J.Q. (2013). Parkinson's disease dementia: convergence of α-synuclein, tau and amyloid-β pathologies. Nat Rev Neurosci.

[38] Pletnikova O., West N., Lee M.K. (2005). Abeta deposition is associated with enhanced cortical alpha-synuclein lesions in lewy body diseases. Neurobiol Aging.

