# Supplementary material for: Distinct CSF α-synuclein aggregation profiles associated with Alzheimer's disease phenotypes and MCI-to-AD conversion
Source: J Prev Alzheimers Dis. 2025 Jan 3;12(2):100040. doi: 10.1016/j.tjpad.2024.100040 (PMC12184058; doi:10.1016/j.tjpad.2024.100040)
Supplement: Supplementary file 1 [file mmc1.docx]

**Supplement Table1. Characteristics of MCI non-convertors and convertors in the follow-up**

|  | **MCI-nc**  **(n=34)** | **MCI-c**  **(n=48)** | ***p* value** |
| --- | --- | --- | --- |
| Age(y) | 73.78 (6.70) | 73.41 (7.92) | 0.828 |
| Gender |  |  |  |
| Female | 12 | 17 | 0.991 |
| Male | 22 | 31 |  |
| Education duration(y) | 15.50 (2.92) | 15.38 (2.97) | 0.850 |
| ApoE ε4 carrier |  |  |  |
| (+) | 16 | 29 | 0.231 |
| (-) | 18 | 19 |  |

**Abbreviations:** MCI: mild cognitive impairment; nc: nonconverters; c: converters.

**Supplement Table 2. Evaluation between the convertors and non-convertors**

|  | **MCI-nc**  **(n=34)** | **MCI-c**  **(n=48)** | **HR**^a^ **(95% CI)** | ***p***^a^ |
| --- | --- | --- | --- | --- |
| Alpha-synuclein |  |  |  |  |
| 0 | 27 | 28 | 2.890  (1.349-7.365) | **0.040** |
| 1 | 7 | 20 |  |  |
| Aβ42, pg/ml |  |  |  |  |
| <730.0 | 16 | 33 | 2.575 | **0.045** |
| ≥730.0 | 18 | 15 | (1.679-6.133) |  |
| p-tau, pg/ml |  |  |  |  |
| <29.0 | 23 | 15 | 4.300  (1.173-10.845) | **0.002** |
| ≥29.0 | 11 | 33 |  |  |
| T-tau, pg/ml |  |  |  |  |
| <310.0 | 23 | 20 | 3.132  (1.415-7.224) | **0.017** |
| ≥310.0 | 11 | 29 |  |  |
| ApoE ε4 carrier |  |  |  |  |
| (+) | 16 | 29 | 1.630  (0.675-3.941) | 0.077 |
| (-) | 18 | 19 |  |  |

a: All results were adjusted for age, education and sex.

**
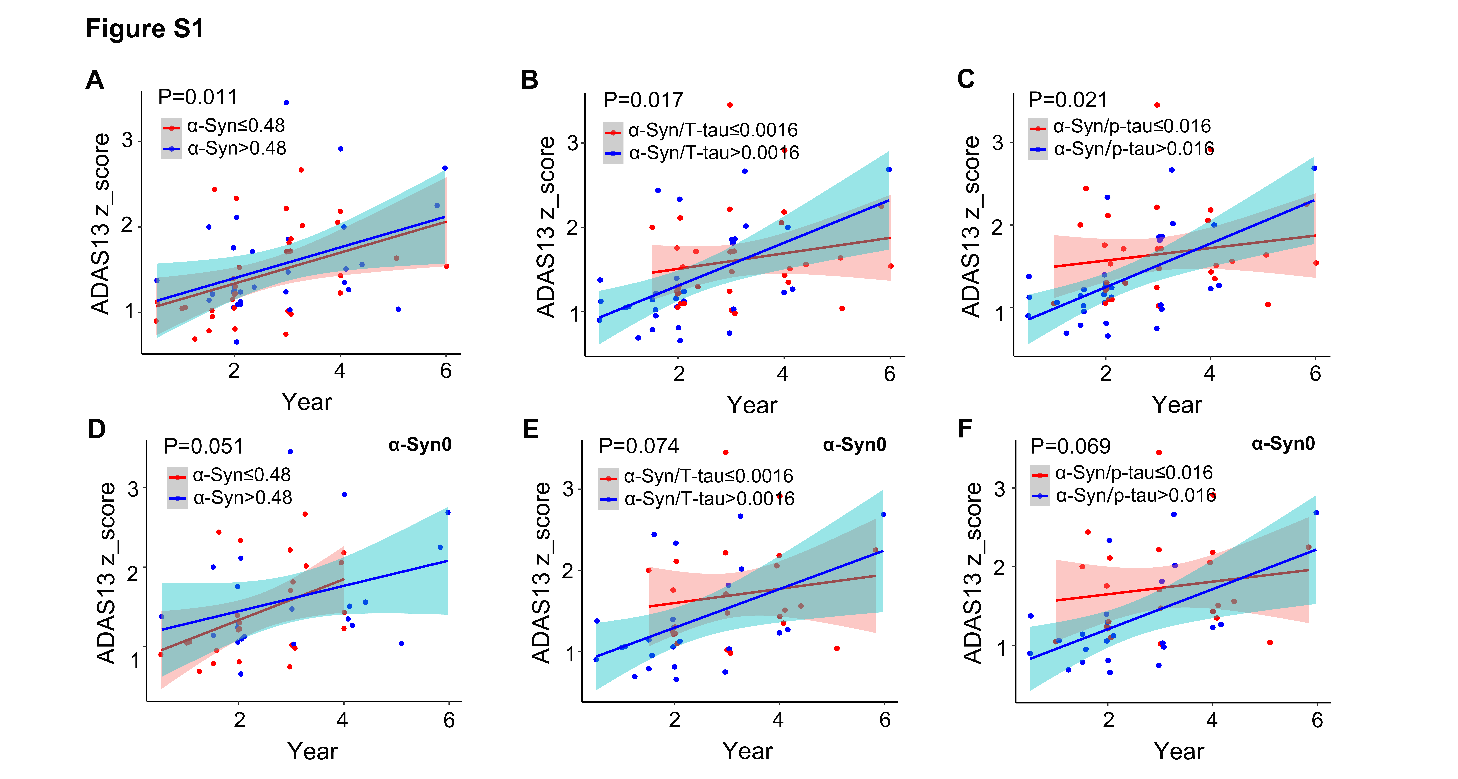
**

**Figure S1: Correlation analysis of CSF α-Syn with cognitive decline in AD patients.**

**A:** Patients with CSFα-Syn levels higher than 0.48 pg/ml experienced faster cognitive impairment. **B:** Patients with CSFα-Syn/T-tau ratio higher than 0.0016 experienced faster cognitive impairment. **C:** Patients with CSFα-Syn/p-tau ratio higher than 0.016 experienced faster cognitive impairment. **D, E, F: In the** α-Syn0 group, α-Syn levels, the ratio of α-Syn/T-tau and α-Syn/p-tau did not correlate with cognitive decline. The cut-off values were determined by using the ROC curve with Youden's index. Multivariable analyses with the Cox proportional hazards model.

**
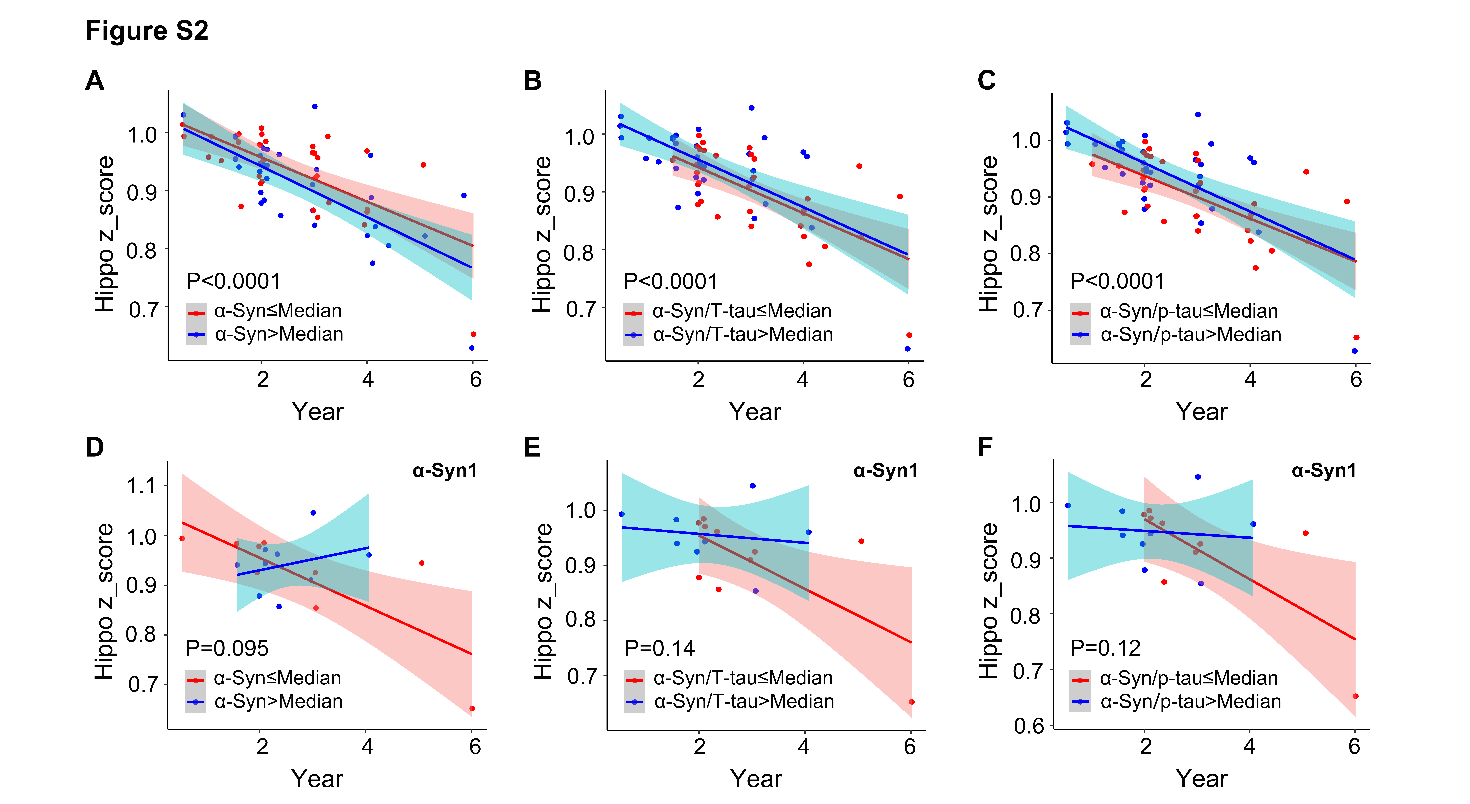
**

**Figure S2: Correlation analysis of CSF α-Syn with hippocampal volume changes in AD patients.**

**A:** A high level ofα-Syn was associated with a faster decline in hippocampal volume. **B:** A high ratio of CSFα-Syn/T-tau was associated with decreased hippocampal volume. **C:** A high ratio of CSFα-Syn/p-tau was associated with decreased hippocampal volume. **D, E, F:** In the α-Syn1 group, the α-Syn levels, the ratio of α-Syn/T-tau and α-Syn/p-tau did not correlate with hippocampal volume changes. Multivariable analyses with the Cox proportional hazards model.
